# The effects of litter input and increased precipitation on soil microbial communities in a temperate grassland

**DOI:** 10.3389/fmicb.2024.1347016

**Published:** 2024-04-08

**Authors:** Xiuli Gao, Zhirong Zheng, Zhaoyan Diao, Yeming Zhang, Yupei Wang, Linna Ma

**Affiliations:** ^1^Institute of Ecology, Chinese Research Academy of Environmental Sciences, Beijing, China; ^2^State Key Laboratory of Vegetation and Environmental Change, Institute of Botany, The Chinese Academy of Sciences, Beijing, China; ^3^International Economics and Trade, University of Technology, Beijing, China

**Keywords:** litter input, increased precipitation, soil microbial community, microbial diversity, microbial biomass, temperate grassland

## Abstract

Global warming has contributed to shifts in precipitation patterns and increased plant productivity, resulting in a significant increase in litter input into the soils. The enhanced litter input, combined with higher levels of precipitation, may potentially affect soil microbial communities. This study aims to investigate the effects of litter input and increased precipitation on soil microbial biomass, community structure, and diversity in a temperate meadow steppe in northeastern China. Different levels of litter input (0%, +30%, +60%) and increased precipitation (0%, +15%, +30%) were applied over a three-year period (2015–2017). The results showed that litter input significantly increased the biomass of bacteria and fungi without altering their diversity, as well as the ratio of bacterial to fungal biomass. Increased precipitation did not have a notable effect on the biomass and diversity of bacteria and fungi, but it did increase the fungal-to-bacterial biomass ratio. However, when litter input and increased precipitation interacted, bacterial diversity significantly increased while the fungal-to-bacterial biomass ratio remained unchanged. These findings indicate that the projected increases in litter and precipitation would have a substantial impact on soil microbial communities. In energy-and water-limited temperate grasslands, the additional litter inputs and increased precipitation contribute to enhanced nutrient and water availability, which in turn promotes microbial growth and leads to shifts in community structure and diversity.

## 1 Introduction

In recent years, human activities such as land use changes and the burning of fossil fuels have resulted in the release of greenhouse gases, leading to global warming (Nielsen and Ball, 2015). This increase in global temperatures has several effects, one of which is an increase in net primary productivity (Xiao et al., 2015; Smith et al., 2016). This means that more organic matter is being produced in terrestrial ecosystems (Schlesinger and Andrews, 2000). Additionally, the Intergovernmental Panel on Climate Change (IPCC) has predicted that global warming will cause shifts in precipitation patterns. In this century, there has been a 7–12% increase in precipitation in the high-and middle-latitude regions of the northern hemisphere (Stocker, 2013; Sun L. et al., 2023). Climate models further suggest that annual precipitation could increase by 30–100 mm (Ni and Zhang, 2000; Xuejie et al., 2001). These changes in litter inputs and increased precipitation are expected to have significant effects on soil microbial communities, including changes in their diversity, biomass, and community structure (Yao et al., 2022). However, our understanding of the specific impacts of litter inputs and increased precipitation on microbial communities in temperate ecosystems remains limited.

Soil microbial communities play important roles in regulating soil processes in terrestrial ecosystems (Chen et al., 2016). Changes in litter inputs, which are the organic materials that accumulate on the soil surface, are closely associated with shifts in microbial communities (Drenovsky et al., 2004; Sylvia, 2005). Litter inputs release nutrients into the soil, providing a food source for soil microbes (David, 2014). In nutrient-limited regions, high soil microbial diversity and biomass are positively correlated with increased litter inputs. This is because most soil microbes, including bacteria and fungi, rely on the decomposition of litter to obtain energy and nutrients (Sniegocki et al., 2022). For instance, the addition of litter has been shown to substantially enhance soil microbial biomass C and total phospholipid fatty acids (PLFAs), indicating an overall increase in soil microbial biomass (Pan et al., 2018). However, the effects of enhanced litter inputs on microbial species may vary, as different microbial species exhibit different growth rates and nutrient utilization abilities (Nadelhoffer et al., 2004; Zhao et al., 2016).

The relationship between soil moisture and microbial communities is complex and can be influenced by various factors, such as soil texture, pH, and depth (Evans and Wallenstein, 2012; Liu et al., 2018). In arid and semi-arid regions, water availability plays a dominant role in shaping soil microbial communities (Sun Q. et al., 2023). Generally, higher water availability in these regions leads to increased soil microbial diversity and biomass (Collins et al., 2008; Curtin et al., 2012). Furthermore, water availability can also affect soil nutrient levels, thereby influencing the growth of specific microbial groups (Avrahami et al., 2003; Yan et al., 2015; Guo et al., 2022). Consequently, changes in precipitation patterns can have significant impacts on soil microbial communities in temperate ecosystems. Further research is needed to fully understand the consequences of these changes and their implications for ecosystem functioning.

The simultaneous occurrence of enhanced litter input and increased precipitation could have the potential to contribute to complex interactions that impact soil microbial communities. Previous studies have shown that, in moist environments, the soil fungal-to-bacterial ratio remains consistently lower, regardless of the amount of litter input. However, in relatively drier conditions, enhanced litter input can lead to a higher soil fungal-to-bacterial ratio (Drenovsky et al., 2004; McIntyre et al., 2009). This suggests that the effect of litter input and water addition on the fungal-to-bacterial ratio may depend on the moisture availability in the environment. However, the detailed mechanisms underlying the interactions between litter input, water addition, and their impact on soil microbial communities are not yet fully understood. Gaining a better understanding of these dynamics is crucial for predicting and managing the responses of soil microbial communities to changing environmental conditions and implementing effective conservation strategies.

It is well known that nutrient and water limitations are prevalent in temperate grasslands, which cover approximately 40% of Chinese terrestrial ecosystems (Kang et al., 2007). Therefore, it is important to investigate the effects of litter input and increased precipitation on soil microbial communities in temperate grassland ecosystems. Based on previous studies, we hypothesize that: (1) increased litter inputs would enhance the diversity and biomass of microorganisms. This is supported by research showing that litter inputs can provide additional nutrients and organic matter, creating favorable conditions for microbial growth (Delgado-Baquerizo et al., 2013; Wang et al., 2014); (2) increased precipitation would also have positive effects on the diversity and biomass of microorganisms. Higher water availability can promote microbial activity and nutrient availability, leading to increased microbial diversity and biomass (Fanin et al., 2014); (3) there would be significant interactive effects between litter input and increased precipitation on microbial communities. This is because increased litter inputs may have different effects under different precipitation levels, as water availability can influence the rate of litter decomposition and nutrient release (Allison et al., 2013; Matulich et al., 2015). These interactive effects may lead to non-additive responses in microbial communities.

## 2 Materials and methods

### 2.1 Study site

In this study, the experiment was conducted in a temperate meadow steppe dominated by *Stipa baicalensis* at the Inner Mongolia Grassland Ecosystem National Field Scientific Observation Station of the Chinese Academy of Agricultural Sciences (49°19′-49°20′N, 119°55′-120°02′E, elevation 628–649 m), situated within Hulunber Grassland in China. The Hulunber Grassland enclosure was established in 1999. The region experiences a continental climate, with a mean average temperature of approximately −1.6°C. The mean average yearly precipitation is about 400 mm, mainly concentrated from June to August. The soil type is classified as chestnut according to Chinese classification or Haplic Calcisol according to the FAO (Food and Agriculture Organization) classification. Perennial grasses, perennial forbs, and shrubs contribute 27, 58, and 5%, respectively, to the aboveground biomass.

### 2.2 Experimental design

In May 2015, a completely randomized block design was used for the experiment (Supplementary Figure S1). The design included four treatments: control, litter input, increased precipitation, and combined litter input and increased precipitation. Each treatment was replicated six times, resulting in a total of 54 plots. Due to the fact that the aboveground biomass in this region was estimated to be around approximately 200 g m^−2^ yr.^−1^ (Evans and Wallenstein, 2012; Ma et al., 2013), the litter addition rates were set at 0 (control), 60 g m^−2^ (+30%), and 120 g m^−2^ (+60%). Each plot had dimensions of 2 × 2 m^2^ and was spaced 2 m apart.

During each spring season, fresh litter was collected from a 100 × 100 m^2^ area adjacent to the experimental plots. The collected litter was carefully cleared of any non-plant debris and air-dried for subsequent use as litter addition material. This approach ensured the consistency of the added material and the plant species present in the area, providing an accurate simulation of litter input scenarios. The organic C, total N, and total P levels of the litter material were 41.26, 0.30, and 0.027%, respectively. To simulate increased precipitation, iron boxes with a base area equivalent to 15% of the plot area (85 cm in length, 71.5 cm in width, and 15 cm in height) were fixed outside the increased precipitation plots to collect natural precipitation. A 1.5 cm hole was carefully created on the side of the iron box, precisely positioned to face the plot. A 2 m tube was securely attached to the hole. To ensure efficient and uniform precipitation distribution within the plots during rainfall events, the tube was strategically arranged in a zigzag pattern across the plot surface. To further enhance the flow of precipitation, small holes were evenly spaced 3 cm apart along the length of the tube (Supplementary Figure S1).

### 2.3 Soil sampling

Soil samples were collected from each plot by randomly obtaining three soil cores (5 cm inner diameter, 10 cm length) from the topsoil (0–10 cm). This sampling procedure was conducted three times throughout the year: in late May, mid-July, and mid-September of both 2016 and 2017. To ensure the representativeness of the samples, the three replicates were mixed together to create a composite sample. The composite sample was then sieved using a 2 mm sieve. From each composite sample, two subsamples of the sieved soil were obtained. One subsample was stored in a refrigerator at 4°C for routine analyses, while the other subsample was kept at −80°C for phospholipid fatty acids (PLFAs) and soil DNA analyses.

### 2.4 Soil microclimate and nutrient measurements

Soil temperature and soil water content measurements were conducted within three days following rainfall events. Soil water content was determined by oven-drying samples at 105°C for 24 h. Soil temperature at a depth of 15 cm was measured by an ECH_2_O sensor (Em50, Decagon) between 8:00 and 9:00 in both 2016 and 2017. Soil NH_4_^+^-N and NO_3_-N concentrations were determined using a flow injection autoanalyzer (FIAstar 5,000 Analyzer, Foss Tecator, Denmark) as described by Ma et al. (2015). Soil microbial biomass C was measured using the fumigation-extraction method and calculated according to the formula described by Wu et al. (1994) and Rotbart et al. (2017).


MBC=EC×Kec

where *EC* is the difference between *C* fumigated and unfumigated soil samples, *Kec* is the conversion factor.

### 2.5 Soil microbial community analysis

To extract the phospholipid fatty acids (PLFAs) from the soil samples, we followed the procedure described by Bossio and Scow (1998). The separation and identification of the PLFAs were performed using the standard protocol of the Sherlock Microbial Identification System V4.5 (MIDI) in combination with a Gas Chromatograph (Agilent 6850, United States). During the analysis, methyl nonadecanoate fatty acid (19:0) was used as the internal standard. For the characterization of the soil microbial community, specific fatty acids were selected to represent different microbial groups. The fatty acids a13:0, i14:0, i15:0, i16:0, i17:0, and a17:0 were chosen to represent gram-positive bacteria. The fatty acids 16:1ω7c, 17:1ω8c, 18:1ω5c, 18:1ω9t, 17:0cy, and 19:0cy were selected to represent gram-negative bacteria. Additionally, three fatty acids, namely 16:1ω5c, 18:2ω6,9c, and 18:1ω9c, were chosen to represent the fungal group (Olsson et al., 1998).

### 2.6 Soil microbial diversity analysis

Genomic DNA was extracted from soil samples using the PowerSoil DNA Isolation Kit from Mo Bio Laboratories. The extracted DNA underwent purity and quality by running it on 0.8% agarose gels. For analysis, the Illumina MiSeq PE300 platform was used. To assess the diversity and composition of soil bacteria and fungi, the V3-4 hypervariable regions of the bacterial 16S rRNA gene were amplified using the primers 806R and 338F. The region of fungal ITS was amplified using the primers ITS1F and ITS2, as described by Caporaso et al. (2012). To ensure unique identification of each soil sample, a 10-digit barcode sequence was appended to the 5′ end of both the forward and reverse primers. PCR amplification was carried out using a Mastercycler Gradient from Eppendorf. For further details on the PCR protocol, refer to the study conducted by Ma et al. (2022).

The raw data underwent quality control procedures, including the removal of sequences with low-quality scores, ambiguous bases, short lengths, and mismatches with primers and barcodes. The processed dataset was analyzed using QIIME, and OTUs were generated at a similarity threshold of 97% to investigate microbial diversity indices. Taxonomic groups were assigned using the Ribosomal Database Project Classifier tool. The OTU datasets were subsampled to compare bacterial and fungal diversity at the same sequencing depth. The number of OTUs served as an indicator of soil microbial taxonomic diversity (Caporaso et al., 2012; Ma et al., 2022).

### 2.7 Statistical analysis

To evaluate the effects of temporal variations (inter-or intra-annual) in litter input and increased precipitation on soil microclimate, inorganic N, soil microbial biomass C, and microbial community, multivariate analysis of variance (MANOVAs) was employed. To further analyze the differences between the means of the effects, multiple comparisons were conducted using the least significant difference test with a significance level of *p* < 0.05. All data management and statistical analyses were performed using SPSS 21.0 software (SPSS, Chicago, United States). Principal Component Analysis (PCA) was employed to analyze the changes in soil microbial community structure, estimated by the concentrations of phospholipid fatty acids (PLFAs), in response to litter input and increased precipitation. The PCA analysis was conducted using R statistical software (v3.3.1, R Core Team, 2016).

## 3 Results

### 3.1 Soil conditions

**Figure 1:**
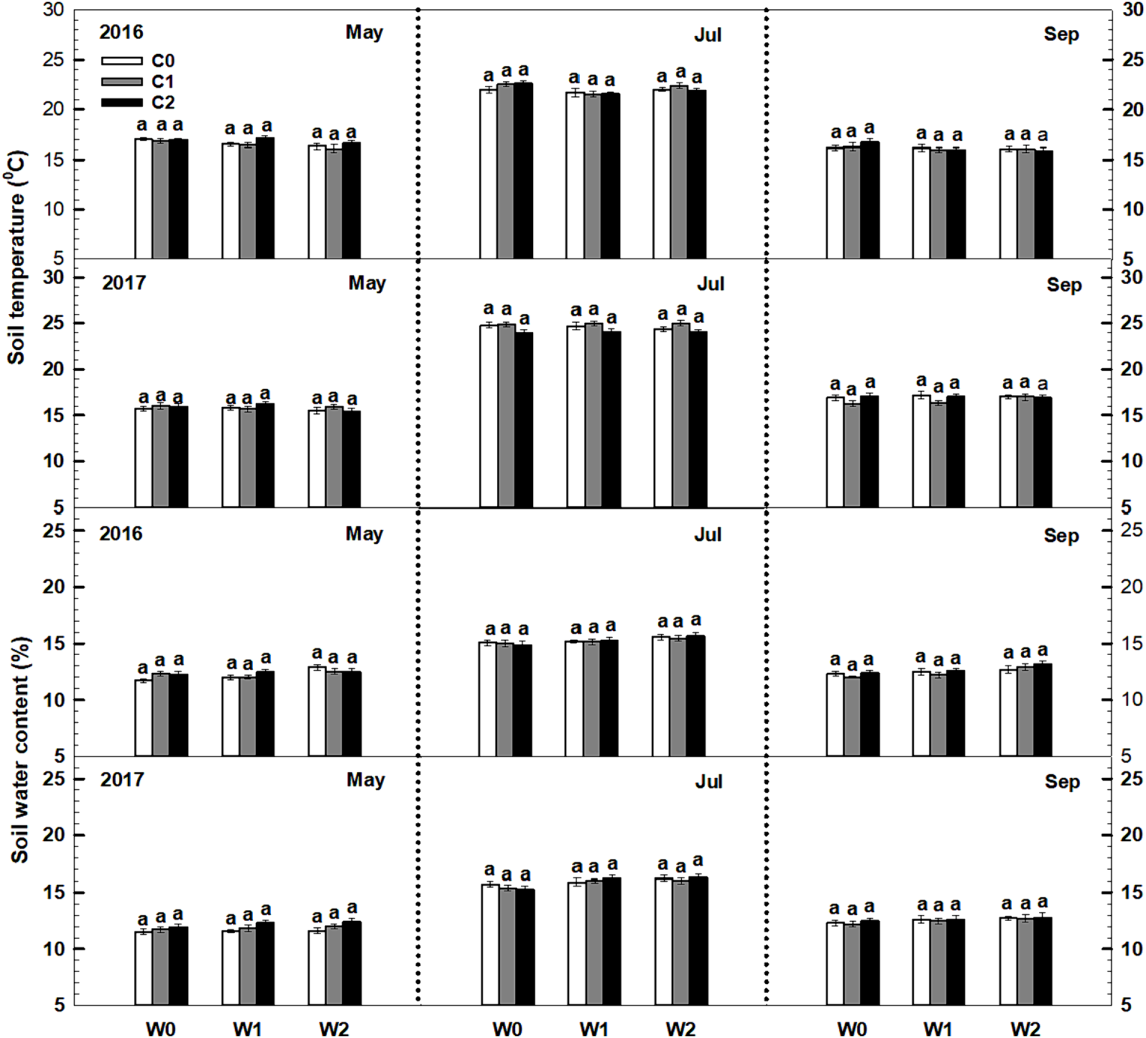
Responses of soil temperature and soil water content to litter input and increased precipitation during the growing seasons in 2016 and 2017. Vertical bars indicate standard errors of means (*n* = 6). C0: control; C1: 30% litter input; C2: 60% litter input; W0: control; W1: increased 15% precipitation; W2: increased 30% precipitation.

Throughout the 2016 and 2017 growing seasons, both soil temperature and soil water content exhibited significant seasonal variations. However, low and high litter inputs did not significantly alter soil temperature and water content (Figure 1). The influence of low and high precipitation additions on soil temperature was also not statistically significant. The interaction of litter input and increased precipitation had no significant impact on soil temperature. Furthermore, no significant interactive effects were observed among sampling time, year, and litter addition (Supplementary Table S1).

**Figure 2:**
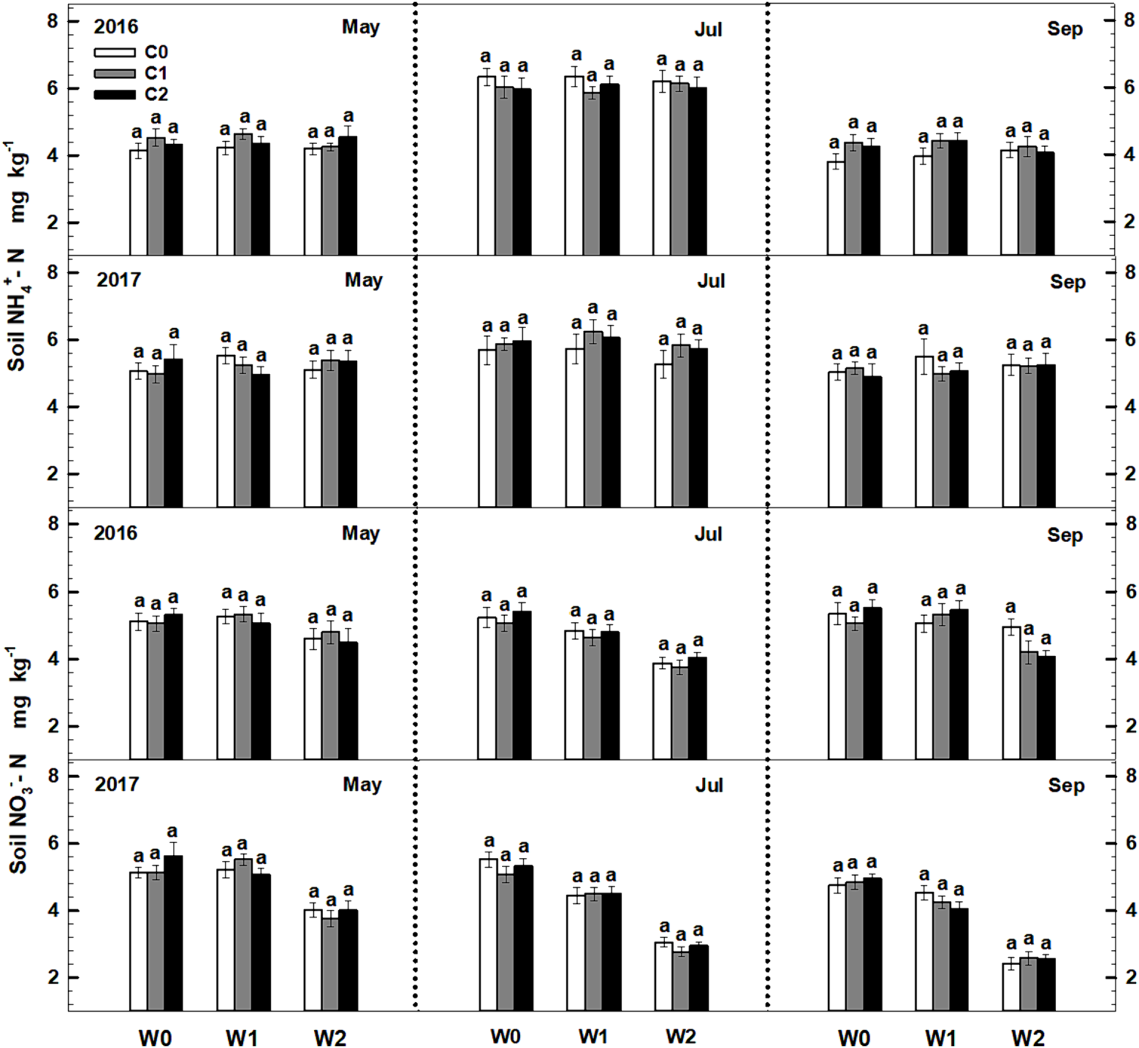
Responses of soil ammonium (NH_4_^+^-N) and nitrate (NO_3_^−^-N) contents to litter input and increased precipitation in 2016 and 2017. Vertical bars indicate standard errors of means (*n* = 6). C0: control; C1: 30% litter input; C2: 60% litter input; W0: control; W1: increased 15% precipitation; W2: increased 30% precipitation.

Significant seasonal variations were observed in soil ammonium content, whereas soil nitrate content remained constant. Both litter input and increased precipitation had no significant impact on the levels of soil ammonium and nitrate. Furthermore, the litter input did not exert any significant effect on soil ammonium and nitrate contents. Furthermore, no interactive effects were observed among sampling time, litter addition, and precipitation addition (Figure 2, Supplementary Table S2).

### 3.2 Soil microbial biomass

**Figure 3:**
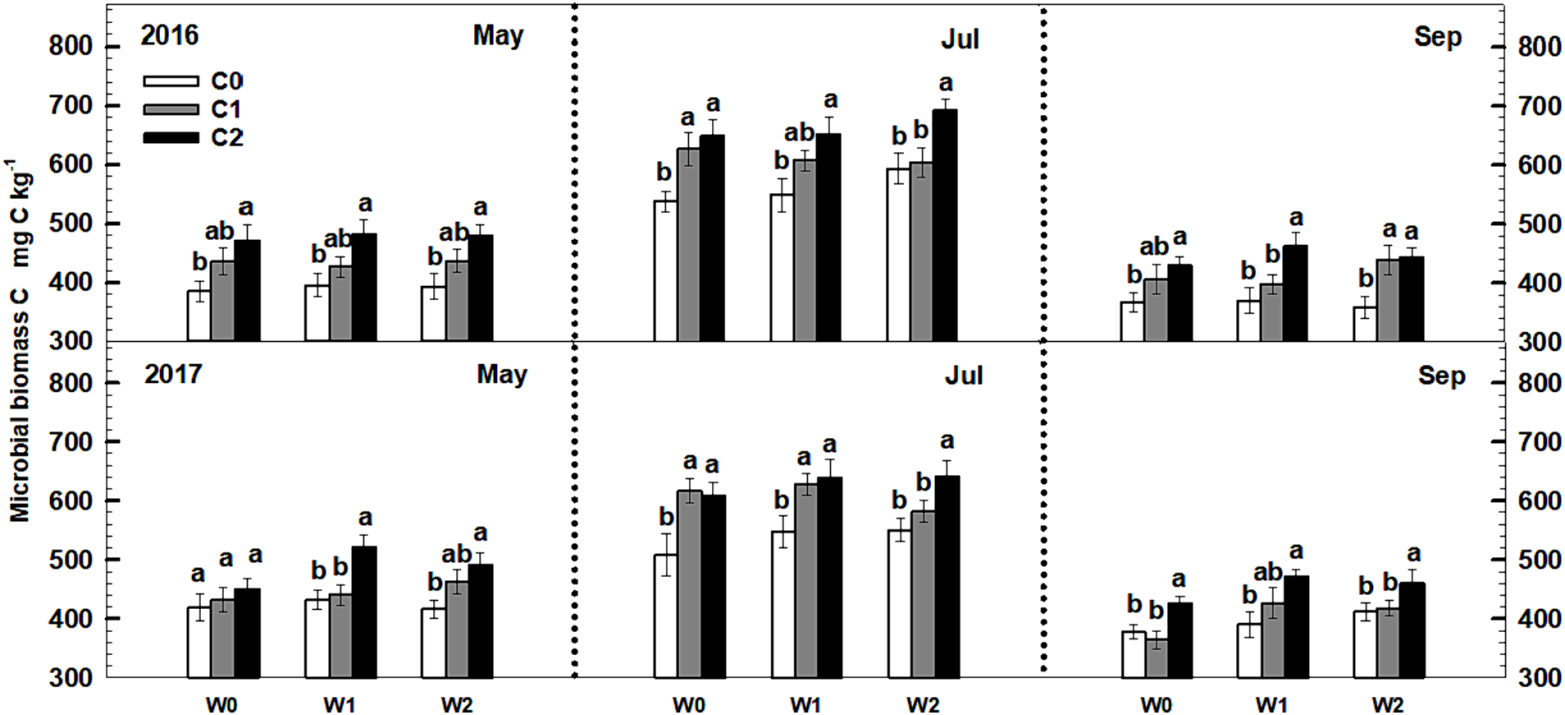
Responses of soil microbial biomass C to litter input and increased precipitation during 2016–2017. Vertical bars indicate standard errors of means (*n* = 6). C0: control; C1: 30% litter input; C2: 60% litter input; W0: control; W1: increased 15% precipitation; W2: increased 30% precipitation.

Soil microbial biomass C exhibited a notable increase with increasing litter input. In 2016, both low and high litter inputs resulted in 12% (*p* < 0.1) and 22.8% (*p* < 0.05) increases in microbial biomass C (Figure 3). Similarly, low and high litter inputs led to 12.4 and 28.1% (*p* < 0.05) increases in microbial biomass C in 2017. However, neither low nor high increased precipitation had a significant impact on soil microbial biomass C. Furthermore, the interactive effects of litter input and increased precipitation did not significantly affect soil microbial biomass C. Neither sampling time nor year displayed significant interactive effects with litter input and increased precipitation treatments on soil microbial biomass C (Supplementary Table S2).

### 3.3 Changes in soil fungal and bacterial PLFAs

**Figure 4:**
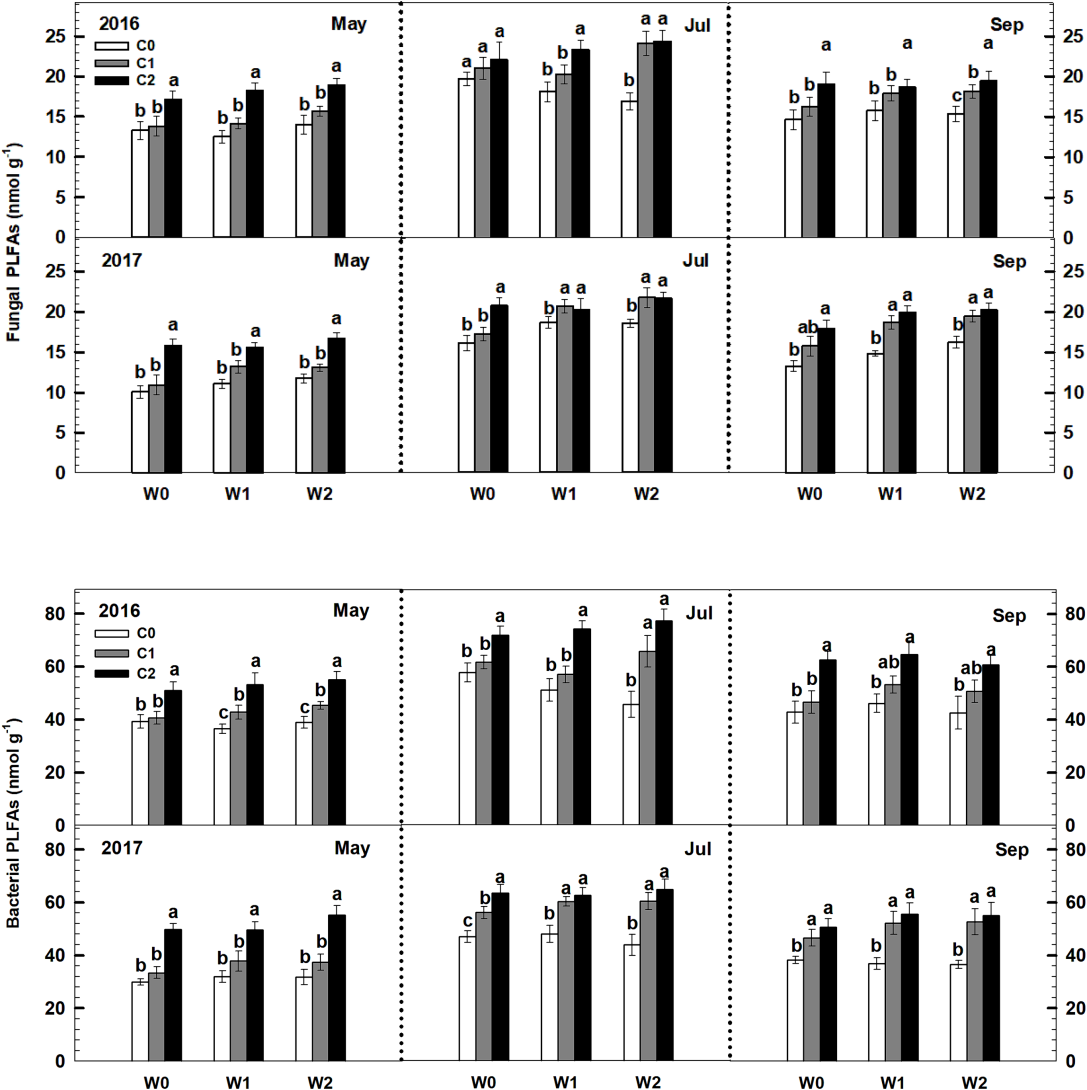
Responses of soil fungal and bacterial PLFAs to litter input and increased precipitation during 2016 and 2017. Vertical bars indicate standard errors of means (*n* = 6). C0: control; C1: 30% litter input; C2: 60% litter input; W0: control; W1: increased 15% precipitation; W2: increased 30% precipitation. Difference lowercase letters indicate statistically significant differences (*p* < 0.05).

Both low and high litter inputs were found to significantly increase soil bacterial PLFAs and fungal PLFAs, indicating an increase in microbial biomass. However, low and high increased precipitation did not have an effect on bacterial PLFAs and fungi PLFAs (Figure 4). Notably, there were significant interaction effects between litter input and increased precipitation on soil bacterial PLFAs and fungal PLFAs (*p* < 0.05, Supplementary Table S3). Interestingly, when litter input and increased precipitation interacted, the ratio of soil fungal to bacterial PLFAs remained unchanged.

**Figure 5:**
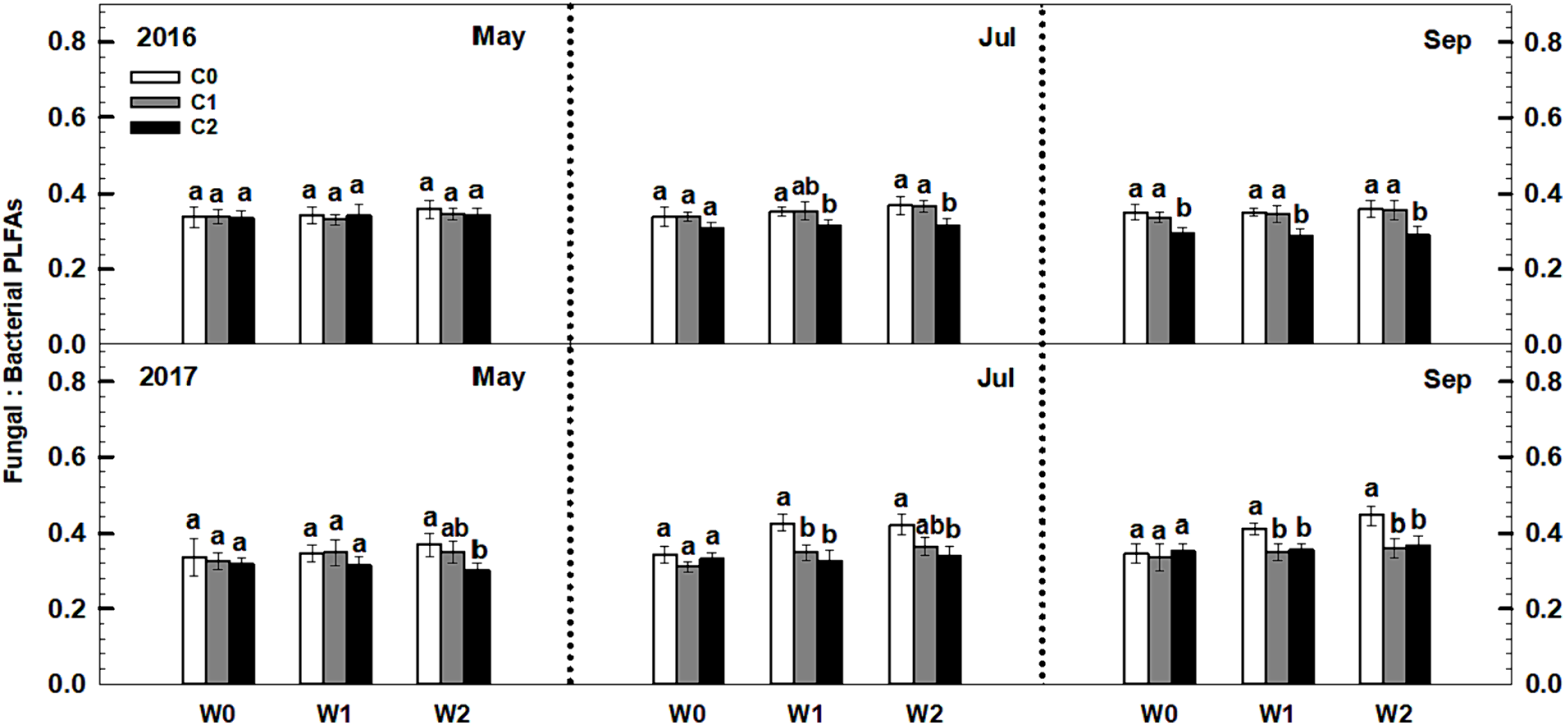
Responses of the ratios of soil fungal to bacterial PLFAs to litter and precipitation additions during 2016 and 2017. Vertical bars indicate standard errors of means (*n* = 6). C0: control; C1: 30% litter input; C2: 60% litter input; W0: control; W1: increased 15% precipitation; W2: increased 30% precipitation. Difference lowercase letters indicate statistically significant differences (*p* < 0.05).

Litter input had no effect on the soil fungal-to-bacterial PLFAs ratio (i.e., fungal-to-bacterial biomass ratio), whereas increased precipitation significantly increased the soil fungal-to-bacterial PLFAs ratio (Figure 5). Notably, an interaction effect was observed between litter input and increased precipitation on soil fungal-to-bacterial PLFAs (*p* < 0.05, Supplementary Table S3). Furthermore, sampling time and year displayed significant interactive effects with litter input and increased precipitation treatments on soil bacterial PLFAs and fungal PLFAs (Supplementary Table S3).

### 3.4 Changes in gram-positive and gram-negative bacterial PLFAs

**Figure 6:**
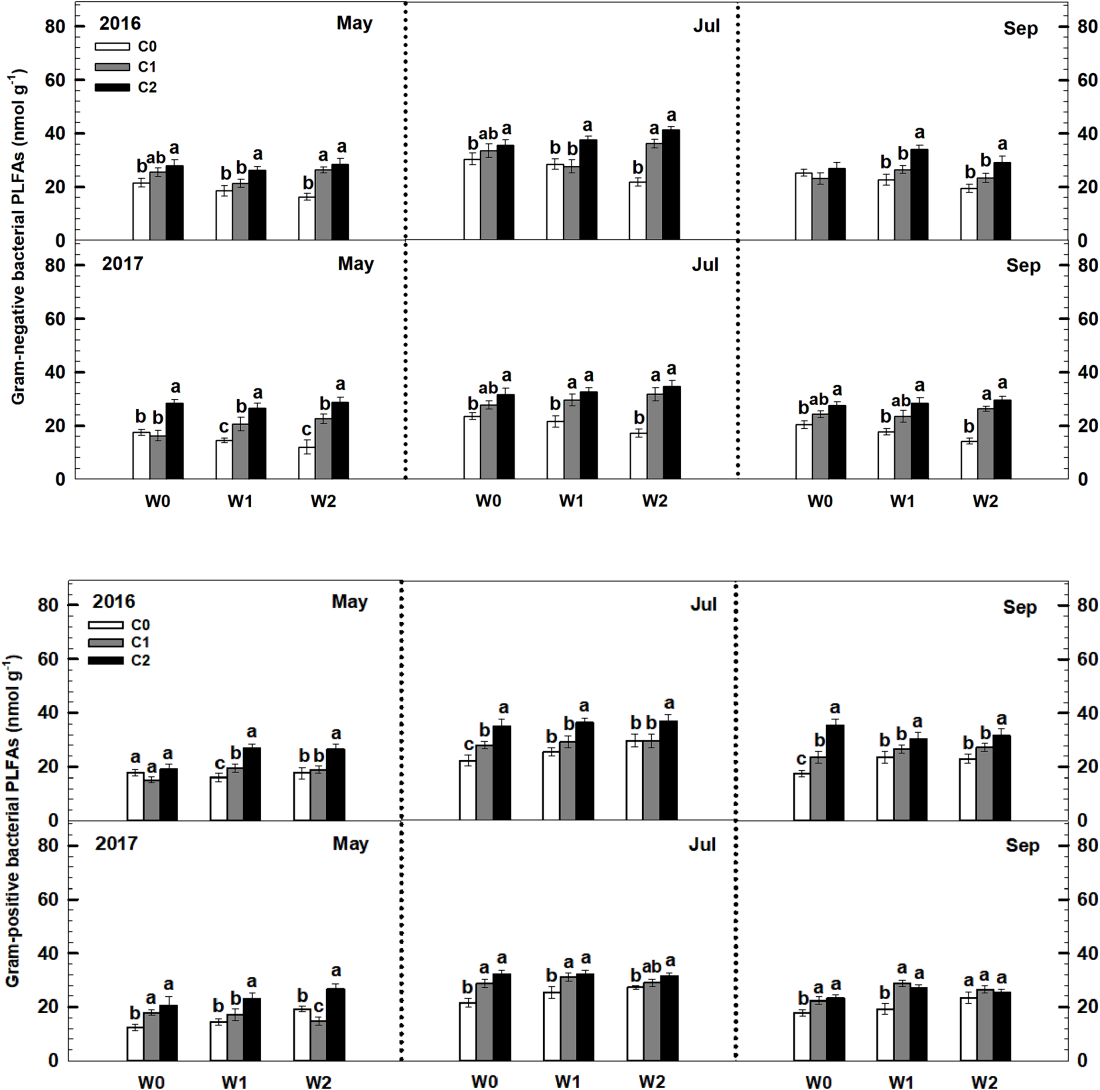
Responses of soil gram-negative bacterial and gram-positive bacterial PLFAs to litter and precipitation additions during 2016 and 2017. Vertical bars indicate standard errors of means (*n* = 6). C0: control; C1: 30% litter addition; C2: 60% litter addition; W0: control; W1: 15% precipitation addition; W2: 30% precipitation addition. Difference lowercase letters indicate statistically significant differences (*p* < 0.05).

Low and high litter inputs significantly increased gram-negative bacterial PLFAs, while they did not affect gram-positive bacterial PLFAs (Figure 6). In contrast, increased precipitation had a significant effect on elevating gram-positive bacterial PLFAs, but it significantly reduced gram-negative bacterial PLFAs. Importantly, there was a significant interaction effect between litter and precipitation additions on both gram-positive bacterial and gram-negative bacterial PLFAs. Sampling time and year exhibited significant interactive effects linked to litter and precipitation addition treatments on soil gram-positive bacterial and gram-negative bacterial PLFAs (Supplementary Table S4).

### 3.5 Soil microbial community structure

**Figure 7:**
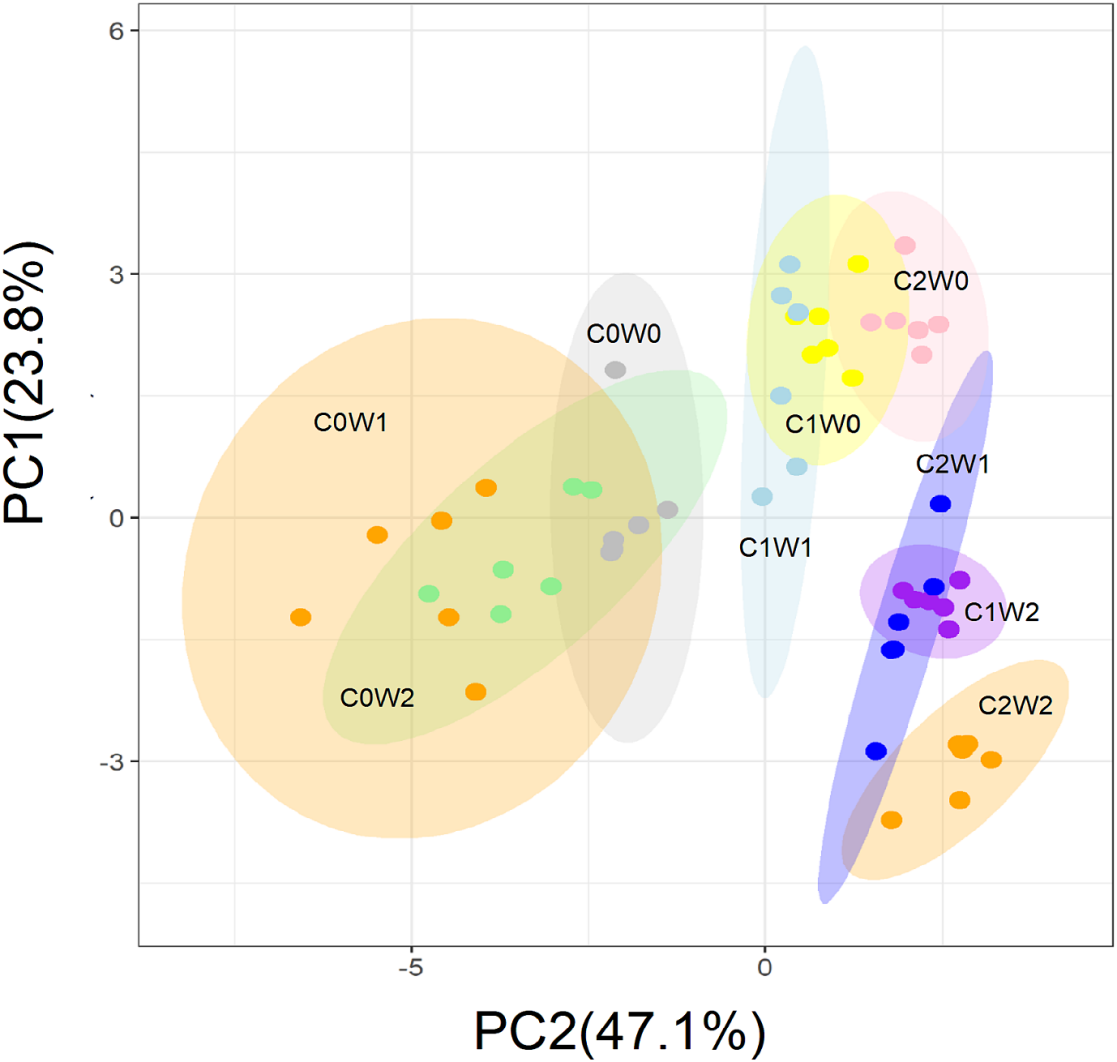
Principal component analysis (PCA) of the variation in the soil microbial community structure estimated by the concentration of the PLFAs under litter and precipitation additions during 2016 and 2017. C0: control; C1: 30% litter addition; C2: 60% litter addition; W0: control; W1: 15% precipitation addition; W2: 30% precipitation addition; PC1: principal component one; PC2: principal component two.

After the three years, principal component analysis (PCA) showed that litter input and increased precipitation altered the soil microbial community structure. The first principal component (PC1) explained 47.1% of the variation in microbial community structure, and the second principal component (PC2) explained 23.8% of the variation (Figure 7). Specifically, PC1 primarily reflected the gradient of litter input, and PC2 represented the gradient of precipitation. Soil bacterial PLFAs, mainly attributed to gram-negative bacteria, and fungal PLFAs showed an increase in response to litter additions. Although the increased precipitation did not have a significant effect on the fungal-to-bacterial ratio, it did lead to an increase in gram-positive bacteria and a decrease in gram-negative bacteria. Notably, when both litter addition and precipitation addition interacted, there was an overall increase in both soil bacterial and fungal PLFAs.

### 3.6 Soil microbial diversity

**Figure 8:**
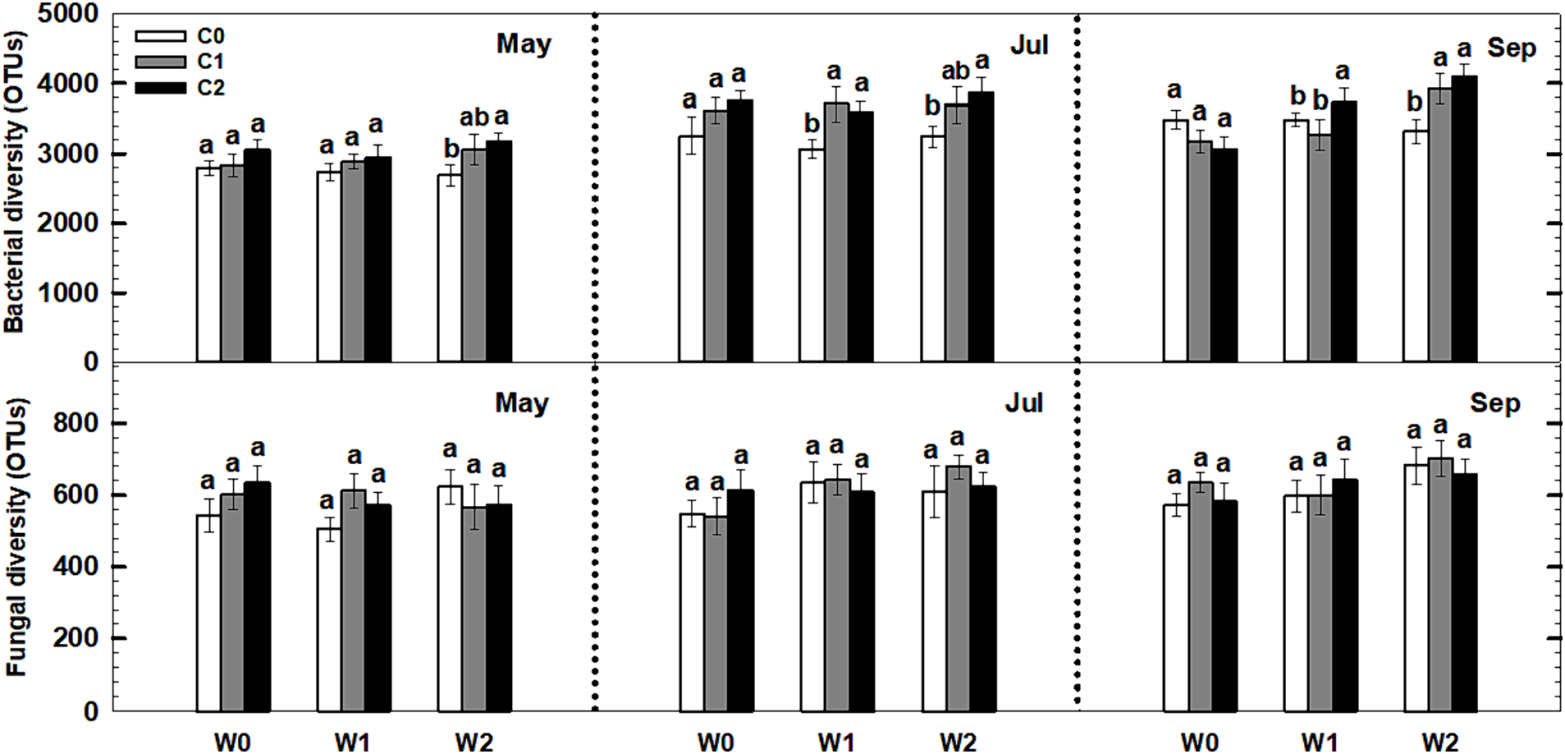
Responses of soil bacterial and fungal diversity to litter input and increased precipitation during 2017. Vertical bars indicate standard errors of means (*n* = 5). C0: control; C1: 30% litter input; C2: 60% litter input; W0: control; W1: increased 15% precipitation; W2: increased 30% precipitation. Difference lowercase letters indicate statistically significant differences (*p* < 0.05).

Neither litter input nor increased precipitation had any effect on the soil bacterial and fungal diversity (i.e., richness) during the third growing season (Figure 8). Notably, an interaction effect was observed between litter input and increased precipitation on soil bacterial diversity (*p* < 0.05, Supplementary Table S5). Furthermore, sampling time and year displayed significant interactive effects with litter input and increased precipitation treatments on soil bacterial diversity and fungal diversity (Supplementary Table S5).

## 4 Discussion

### 4.1 Effect of litter input on soil microbial communities

The findings of this study show that litter addition increased both soil fungal and bacterial biomass in the temperate grassland. This supports previous research that has also found increases in soil microbial biomass following litter addition (Bailey et al., 2002; Creamer et al., 2015). The increase in microbial biomass is likely due to the higher availability of organic C from decomposing litter, which promotes fungal growth. However, contrary to expectations based on previous research, the fungal-to-bacterial biomass ratio did not change significantly (Figure 5). Previous studies have suggested that higher litter accumulation increases this ratio (Bailey et al., 2002; Drenovsky et al., 2004), but the results of this study did not support that hypothesis. However, in the DIRT (Detritus Input Removal and Transfer) experiment, the removal or addition of litter altered the composition of soil microbial communities, leading to shifts in the proportion of soil fungi to bacteria (Siira-Pietikäinen et al., 2003; Nadelhoffer et al., 2004; Brant et al., 2006). In contrast, our results align with the findings of Kirchmann et al. (2004), Morris and Boerner (1999), and Busse et al. (2009), which also reported no significant alteration in microbial community structure despite changes in soil bacterial and fungal communities. The lack of change in the fungal-to-bacterial biomass ratio could be attributed to the complex interactions between litter quality, nutrient availability, and microbial community composition (Fanin et al., 2014). Litter inputs, when accompanied by nutrient enrichment, may alleviate nutrient limitations among microbial groups and help maintain a stable fungal-to-bacterial biomass ratio (Morris and Boerner, 1999). Understanding these dynamics is crucial for predicting the consequences of litter accumulation and decomposition on ecosystem processes and functions.

While the proportion of soil bacterial biomass within the microbial community remained non-significant, gram-negative bacteria in the bacterial community increased with extra litter addition, while the response of gram-positive bacteria biomass to litter addition was not significant (Figure 7). This aligns with the findings of Bossio and Scow (1998) and Zelles et al. (1992). Bossio and Scow (1998) studied the response of soil microbial community structure to substrate additions in agroecosystems, indicating a strong correlation between gram-negative bacterial PLFAs and the availability of soil organic matter. This finding suggests that higher organic matter availability leads to higher gram-negative biomass. Therefore, gram-negative biomass may be a predictor of soil quality in temperate grasslands.

### 4.2 Response of soil microbial communities to increased precipitation

Under the increased precipitation treatments, there was a significant increase in the fungal-to-bacterial ratio in the soil (Figure 6). This shift in the soil microbial community structure can be explained by several factors. Firstly, increased precipitation promotes plant growth, which in turn accelerates the uptake of inorganic N by plants and the leaching of nitrate. As a result, the concentration of inorganic N in the topsoil decreases significantly. This decrease in inorganic N content creates a more nutrient-restricted environment in the temperate grassland, leading to intensified competition between plants and soil microbes for limited N resources (Kuzyakov and Xu, 2013; Liu et al., 2016). Secondly, it is important to note that under nutrient-restricted conditions, soil fungi have a competitive advantage over bacteria (Shao et al., 2022). This advantage stems from the ability of fungal hyphae to extend into different soil layers, extracting the necessary nutrients and accessing a wider range of nutrient sources (Cairney, 2005). Furthermore, fungi possess filamentous characteristics that enable cytoplasmic streaming and nutrient redistribution within their cells, facilitating nutrient recycling (Bonfante and Genre, 2010; Guhr et al., 2015). Consequently, the reduction in inorganic N content resulting from increased precipitation enhances the proportion of fungi in the soil microbial community. This increase in the fungal-to-bacterial biomass ratio highlights the importance of fungal contributions to nutrient cycling and ecosystem functioning in N-limited grassland ecosystems.

Under the increased precipitation treatments, there was a notable shift in the composition of bacterial communities in the soil. Specifically, the biomass of gram-positive bacteria exhibited an increase, while the biomass of gram-negative bacteria presented a significant decrease. This finding is consistent with a previous study at the agricultural field, which revealed that arid soils were characterized by a higher abundance of gram-negative bacteria, whereas relatively moist soils were dominated by gram-positive bacteria (Drenovsky et al., 2004). The observed increase in the biomass of gram-positive bacteria under higher precipitation levels can be attributed to several factors. Firstly, the additional precipitation promotes plant growth, leading to an increased input of organic matter into the soil. Gram-positive bacteria are known to thrive in nutrient-rich environments and are efficient decomposers of organic material. Therefore, the availability of organic matter in the soil, due to increased plant growth, favors the proliferation of gram-positive bacteria (Fanin et al., 2019). Furthermore, the decrease in the biomass of gram-negative bacteria can be explained by changes in nutrient availability and competition for resources. Under nutrient-restricted conditions caused by the decrease in inorganic N content, competition for limited resources intensifies between plants and soil microbes. Gram-negative bacteria, which are more specialized in utilizing inorganic N sources, may face greater competition from plants for these resources. In contrast, gram-positive bacteria have a wider range of nutrient acquisition strategies and can effectively utilize organic matter, giving them a competitive advantage under nutrient-restricted conditions (Francis et al., 2010).

### 4.3 Interactive effect of litter input and increased precipitation on microbial community structure

Currently, there is limited research on the effects of litter and precipitation additions on soil microbial community structure in arid and semi-arid regions (Creamer et al., 2015). The findings of this study indicated that increased precipitation had a significant effect on the ratio of soil fungal to bacterial biomass. However, intriguingly, when increased precipitation was combined with litter addition, there was no notable alteration in the fungal-to-bacterial biomass ratio. This finding suggests that the effect of increased precipitation on microbial community structure is altered when combined with litter input, indicating that the litter effect modulates the precipitation effect in the temperate grassland ecosystem. One possible explanation for this observation is that litter addition, which includes nutrient release, ameliorates nutrient limitations for microbes, thereby attenuating the impact of increased precipitation on microbial community structure.

The combined effect of litter input and increased precipitation may have a greater impact on bacterial diversity compared to their individual effects (Figure 8). There are several potential reasons for this interaction effect. Firstly, both litter input and increased precipitation can influence soil moisture, a crucial factor for microbial diversity. Litter input acts as a mulch layer, reducing evaporation and promoting water availability for microbes (Wang et al., 2016). Increased precipitation directly adds moisture to the soil, potentially creating more favorable conditions for microbial growth. Therefore, the combination of litter input and increased precipitation can result in higher soil moisture levels, enhancing microbial diversity. Secondly, litter input and increased precipitation can provide additional organic matter and nutrients to the soil, which can fuel microbial growth and activity (Ma et al., 2013; Wu et al., 2017). Litter input serves as a source of carbon and other nutrients utilized by bacteria and fungi. Increased precipitation can leach nutrients from litter and other organic materials, making them more accessible to microbial communities. This increased nutrient availability can support the growth of a wider range of bacterial species, resulting in higher diversity. In order to fully understand the underlying mechanisms and long-term effects of these interactions on microbial communities and ecosystem functioning, further research is required.

### 4.4 Future work

Based on the findings mentioned, future research can focus on several aspects to enhance our understanding of the interactions between organic matter, water, and soil microbial communities. Firstly, it is important to explore how organic matter and water influence the functional traits of soil microbial communities. In addition to their impact on microbial community structure, organic matter and water may also alter the functional characteristics of microbial communities, such as their ability to cycle C and N. By investigating these functional changes, we can gain valuable insights into how organic matter and water affect the overall functioning of soil ecosystems. Secondly, it would be beneficial to investigate the effects of different types of organic matter on the structure and function of soil microbial communities. Leaf litter and woody debris, for example, may have distinct impacts on microbial communities due to their unique chemical composition and rates of decomposition. By understanding how different types of organic matter influence microbial communities, we can further elucidate the underlying mechanisms driving these interactions. Lastly, integrating experimental research with field observations can provide insights into the long-term effects and ecological significance of organic matter and water on soil microbial communities. Long-term monitoring and experiments can better simulate and predict the impacts of future climate change on soil microbial communities, and provide scientific basis for ecosystem management and conservation.

## 5 Conclusion

With a three-year field manipulative experiment, the findings showed that litter input significantly increased soil microbial biomass, including bacterial and fungal biomass. However, litter input did not lead to any changes in the soil fungal-to-bacterial biomass ratio or microbial diversity in the temperate grassland. In contrast, increased precipitation had no significant impact on soil microbial biomass and diversity, but it increased the soil fungal-to-bacterial ratio. However, when litter input and increased precipitation interacted, there was a significant increase in bacterial diversity. This suggests that the combination of litter input and increased precipitation can alleviate nutrient limitations on soil microbial growth, leading to an increase in bacterial diversity. This also suggests that litter input moderates the influence of precipitation on soil microbial growth. These findings imply that in water-and nutrient-limited temperate grasslands, the input of litter and precipitation can enhance substrate energy and nutrient levels, which in turn alters soil microbial growth. It is also important to note that these changes in microbial communities have long-term implications for ecosystem dynamics. In the future, as precipitation levels increase and litter accumulation continues, the soil microbial communities are expected to undergo significant changes. Overall, this study provides valuable insights into the complex interactions between litter input, precipitation, and soil microbial communities. These findings can be used as robust parameters for terrestrial ecosystem modeling, contributing to a better understanding of ecosystem dynamics and facilitating effective ecosystem management.

## Data availability statement

The datasets presented in this study can be found in online repositories. The names of the repository/repositories and accession number(s) can be found at: DataDryad – 10.5061/dryad.3tx95x6pc.

## Author contributions

XG: Writing – original draft, Methodology, Investigation, Formal analysis. ZZ: Writing – original draft, Investigation, Funding acquisition, Formal analysis. ZD: Writing – original draft, Methodology, Investigation, Formal analysis. YZ: Writing – original draft, Methodology, Investigation, Formal analysis, Data curation. YW: Writing – original draft, Investigation. LM: Writing – review & editing, Supervision, Conceptualization.
