# Supplementary material for: The effects of litter input and increased precipitation on soil microbial communities in a temperate grassland
Source: Front Microbiol. 2024 Apr 8;15:1347016. doi: 10.3389/fmicb.2024.1347016 (PMC11033436; doi:10.3389/fmicb.2024.1347016)
Supplement: Supplementary file 1 [file Data_Sheet_1.PDF]

Supplementary information

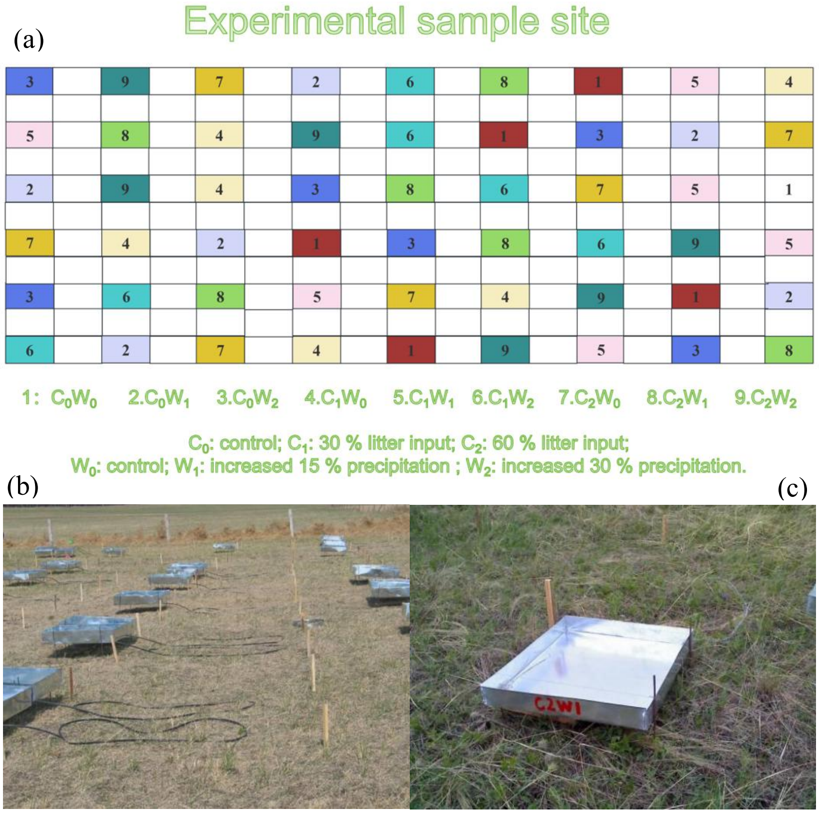

Fig. S1 A completely randomized block design was used in the study site (a; C_0: control; C_1: 30 % litter input; C_2: 60 % litter input; W_0: control; W_1: increased 15 % precipitation ; W_2: increased 30 % precipitation). Pictures of the increased precipitation treatments in this study site (b, c).

Table S1 Results (*F*-value and *P*-value) of multivariate analysis of variance (MANOVAs) on the effects of litter input (C), increased precipitation (W), sampling date (D), year (Y), and their interactions on soil temperature (ST) and soil water content (SWC).

	**ST**		**SWC**	
	F	***P***	***F***	***P***
**C**	0.23	0.352	2.85	0.077
**W**	0.97	0.294	14.73	< 0.001
**C×W**	0.43	0.532	8.85	0.032
**D**	110.63	< 0.001	172.84	< 0.001
**D×C**	0.53	0.352	0.25	0.793
**D×W**	1.05	0.295	1.04	0.275
**D×C×W**	0.62	0.463	0.53	0.698
**Y**	2.04	0.126	12.05	< 0.001
**Y×C**	0.63	0.692	0.55	0.85
**Y×W**	3.05	0.088	2.74	0.207
**Y×C×W**	1.06	0.608	2.05	0.175

Table S2 Results (*F*-value and *P*-value) of multivariate analysis of variance (MANOVAs) on the effects of litter input (C), increased precipitation (W), sampling date (D), year (Y), and their interactions on soil ammonium (NH_4^+-N) N, nitrate N (NO_3^--N) contents, microbial biomass C (MBC).

	NH_4^+-N		NO_3^--N		MBC	
	F	***P***	***F***	***P***	***F***	***P***
**C**	0.88	0.632	1.02	0.077	178.53	< 0.001
**W**	12.85	< 0.001	14.38	< 0.001	2.04	0.131
**C×W**	0.40	0.724	1.03	0.032	1.55	0.096
**D**	126	< 0.001	98.72	< 0.001	352.52	< 0.001
**D×C**	0.77	0.503	0.83	0.793	1.02	0.241
**D×W**	2.17	0.093	1.55	0.275	0.62	0.434
**D×C×W**	0.89	0.862	1.03	0.698	0.55	0.634
**Y**	5.07	0.048	21.26	< 0.001	3.54	0.035
**Y×C**	1.22	0.263	1.46	0.104	0.62	0.724
**Y×W**	1.75	0.391	3.05	0.038	1.04	0.226
**Y×C×W**	2.27	0.246	2.05	0.094	0.86	0.523

Table S3 Results (*F*-value and *P*-value) of multivariate analysis of variance (MANOVAs) on the effects of litter input (C), increased precipitation (W), sampling times (D), year (Y), and their interactions on fungal PLFAs (F), bacterial PLFAs (B) and the ratios of fungal to bacterial PLFAs (F: B).

	**F**		**B**		**F:B**	
	F	***P***	***F***	***P***	***F***	***P***
**C**	104.23	< 0.001	215.53	< 0.001	2.14	0.094
**W**	2.13	0.094	1.85	0.162	8.42	0.017
**C×W**	17.24	0.000	9.55	0.000	10.42	0.005
**D**	152.62	< 0.001	87.25	< 0.001	97.42	< 0.001
**D×C**	3.05	0.063	5.10	0.038	0.69	0.388
**D×W**	1.05	0.083	2.19	0.117	2.45	0.184
**D×C×W**	1.04	0.127	3.52	0.102	1.03	0.225
**Y**	9.24	0.001	10.52	0.003	5.13	0.063
**Y×C**	1.52	0.106	2.04	0.162	2.62	0.227
**Y×W**	1.35	0.078	2.46	0.224	1.52	0.173
**Y×C×W**	1.66	0.226	3.06	0.132	2.72	0.088

Table S4 Results (*F*-value and *P*-value) of multivariate analysis of variance (MANOVAs) on the effects of litter input (C), increased precipitation (W), sampling date (D), year (Y), and their interactions on gram-negative bacterial PLFAs (GN) and gram-positive bacterial PLFAs (GP).

	**GN**		**GP**	
	F	***P***	***F***	***P***
**C**	9.53	0.003	2.05	0.153
**W**	6.87	0.021	7.92	0.015
**C×W**	8.22	0.006	5.73	0.041
**D**	56.82	< 0.001	67.61	< 0.001
**D×C**	8.24	0.001	4.72	0.063
**D×W**	5.33	0.042	4.75	0.073
**D×C×W**	2.73	0.074	5.62	0.092
**Y**	6.66	0.042	7.24	0.036
**Y×C**	4.72	0.073	2.73	0.152
**Y×W**	3.74	0.121	6.24	0.083
**Y×C×W**	9.24	0.001	6.77	0.037

Table S5 Results (*F*-value and *P*-value) of multivariate analysis of variance (MANOVAs) on the effects of litter input (C), increased precipitation (W), sampling time (D), and their interactions on bacterial diversity (BAC) and fungal diversity (FUN).

	**BAC**		**FUN**	
	F	***P***	***F***	***P***
**C**	2.01	0.195	0.95	0.371
**W**	1.42	0.205	1.03	0.195
**C×W**	8.82	< 0.001	1.74	0.184
**D**	21.76	< 0.001	15.82	< 0.001
**D×C**	2.02	0.163	1.73	0.114
**D×W**	1.73	0.206	2.41	0.091
**D×C×W**	7.24	< 0.001	5.82	0.038
